# Trends in fluoroquinolone prescribing in UK primary and secondary care between 2019 and 2023

**DOI:** 10.1093/jac/dkae440

**Published:** 2024-12-21

**Authors:** Fergus Hamilton, Elizabeth Darley, Karon Arnold, Alasdair MacGowan

**Affiliations:** MRC Integrative Epidemiology Unit, University of Bristol, Bristol, UK; Infection Science, North Bristol NHS Trust, Bristol, UK; Infection Science, North Bristol NHS Trust, Bristol, UK; Bristol, South Gloucestershire, and North Somerset Integrated Care Board, Bristol, UK; Infection Science, North Bristol NHS Trust, Bristol, UK

## Abstract

**Introduction:**

Fluoroquinolones are important antibiotics but have associations with a number of adverse outcomes. A recent (January 2024) decision by the UK drug regulator, the Medicines and Health Regulatory Authority (MHRA), restricted systemic use of these antibiotics to when ‘absolutely necessary’. One stated reason for the ban was the failure of previous guidance (2019, 2023) to reduce prescribing, with the MHRA stating there had been ‘no change in prescribing’ of fluoroquinolones in relation to guidance.

**Methods:**

We evaluated the trend in prescribing of fluoroquinolones and comparator antibiotics using national data for all primary care practices in England from 2019 to 2023. We calculated the percent change in prescribing of fluoroquinolones using linear regression, comparing with other antibacterials. We also performed analysis on secondary care prescribing and included hospital inpatient stay data.

**Results:**

In primary care, there was a negative trend in fluoroquinolone item dispensing, with a 4.2% reduction in items dispensed per year (95% CI −5.2% to −3.3%; *P* = 6 × 10^−13^). This occurred despite no change in overall antibacterial prescription and no decrease in comparator antibiotics. Secondary care data showed stable prescription of fluoroquinolones, but comparator antibiotics increased, leading to relatively fewer prescriptions compared with other agents.

**Conclusions:**

There was a reduction in fluoroquinolone prescribing in England in absolute and relative terms between 2019 and 2023 in primary care, and absolute terms in secondary care. These findings do not support the MHRA’s claim that there has been no change in prescribing in response to warnings.

## Introduction

Fluoroquinolones are a widely used class of antibiotics.^1^ They are effective therapy for Gram-negative, Gram-positive and (for some agents) anaerobic bacteria.^1^ The most commonly used agents are ciprofloxacin, levofloxacin, ofloxacin and moxifloxacin, although the first two are far more commonly prescribed. Ofloxacin is generally reserved for treatment of sexually transmitted infection and prostatitis,^2^ while moxifloxacin is rarely used due to QT prolongation.^3^ Given their broad spectrum of *in vitro* activity and generally favourable pharmacokinetics, they are widely recommended across a broad range of infection indications, particularly in patients with penicillin allergy. They were, until very recent announcements from the Medicines and Health Regulatory Authority (MHRA^4^) the recommended option for severe community-acquired pneumonia (CAP) with a penicillin allergy in UK NICE guidance.^5^ They are also recommended in the 2019 IDSA/American Thoracic Society (ATS) pneumonia guidance,^6^ and as first line and/or recommended in penicillin allergy or under other conditions in conditions as broad as prosthetic joint infection, *Legionella* pneumonia,^6^ sexually transmitted infections,^7^ typhoid fever,^8^ pyelonephritis,^9^ TB^10^ and Gram-negative infections including bacteraemia.^11^ For some deep tissue infections, they may be given for a course lasting several weeks or months, and are the preferred therapy. Given the paucity of effective oral agents for Gram-negative infection and high resistance rates to other drugs, fluoroquinolones are highly valuable agents. For infections caused by some organisms such as *Pseudomonas aeruginosa*, they are the only oral option. In current UK practice, the majority of use in primary care is for acute prostatitis or where culture results identify it as the only oral option (e.g. in ESBL-positive urinary tract infection, or pseudomonal exacerbation of bronchiectasis). In secondary care, most usage relates to severe pneumonia, complex urinary tract infection, or step-down therapy from initial broad-spectrum therapy in suspected Gram-negative infection.

Despite these benefits, fluoroquinolones are known to be associated with a range of adverse events.^12^ Well-established and proven causal associations include tendonitis and *Clostridioides difficile* infection, but over the past 20 years, an increasing number of associations have been identified, including QTc prolongation, aortic dissection, aortic aneurysm and (sometimes severe) psychiatric associations.^12^ Regulators have been aware of these risks for some time, with the first FDA ‘black box’ warning in 2008 (related to tendonitis).^13^ More recently the EMA and MHRA have produced multiple updates with increasing concern. The EMA produced guidance suggesting use should be limited to only severe infection in March 2019 (due to the risk of tendonitis and potentially aortic dissection).^14^ The MHRA have also produced a number of updates, starting in 2019,^15^ repeated in 2023,^16^ and most recently, in 2024,^4^ a much tighter restriction of fluoroquinolone prescribing. This directs against any use of systemic fluoroquinolone therapy except where absolutely necessary (e.g. no other drug can be used). The extent of this ban resulted from an ongoing review of the evidence, increasing reports of patient adverse reactions via the national scheme (Yellow Card), and ‘no evidence of a change in fluoroquinolone prescribing patterns’ in relation to previous warnings.^16^ It is clear that this ban surprised clinicians with both its suddenness and the degree of restrictions imposed.^17,18^

The reference for this ‘lack of change’ in prescribing was a European pharmacovigilance study,^19^ which included 15 million UK participants from 2016 to 2020 and was commissioned by the EMA to assess the impact of the 2019 EMA guidance across numerous countries. It was not able to identify a reduction in fluoroquinolone usage directly in relation to the EMA guidance.^19^

However, this study did show that (i) the UK had the lowest rates of fluoroquinolone prescribing in all included countries, and (ii) the UK had around a 3% reduction per year in fluoroquinolone prescriptions from 2016 to 2020 guidance.^19^

Given this study ended during 2020 (when COVID-19 strongly impacted antimicrobial prescribing) and showed an apparent reduction in prescribing, we performed a study of UK fluoroquinolone prescribing in primary care using all of England primary care and secondary care data. Our primary aim was to identify the recent trend in fluoroquinolone prescribing.

## Methods

To evaluate trends in prescribing of fluoroquinolones, we extracted data on England-wide prescribing in both primary and secondary care datasets.

### Primary care dataset

Primary care data were accessed via OpenPrescribing,^20^ which extracts directly from NHS primary care records for England. We extracted specific data on quinolones (as a class), ciprofloxacin and levofloxacin. Fluoroquinolones are the only regularly prescribed quinolones in the UK and so we use the terms interchangeably in this report. Ciprofloxacin accounted for >95% of prescriptions, and no other quinolone agents were prescribed widely.

For comparison, we also extracted data on two comparator agents with similar spectrums of activity: co-amoxiclav and co-trimoxazole. We also extracted total antibiotic use for all penicillins, all antibacterials, and total medication use. Data were available from December 2018 until November 2023 (4 years 11 months), at a monthly level, and at Integrated Care Board (ICB) level. There are 101 ICBs in England. Data are in the format of number of prescriptions and quantity dispensed (as most prescriptions are for more than one dose). We estimated DDDs using BNF dosing (i.e. as ciprofloxacin is dosed twice daily, the quantity divided by two is the DDD).

The DDD is ‘the assumed average maintenance dose per day for a drug used for its main indication in adults’ as defined by the WHO Collaborating Centre for Drug Statistics Methodology (WHOCC).^21^ It provides a fixed unit of measurement for drug consumption irrespective of the different quantities being prescribed for different durations. A DDD is established for each drug coded in the Anatomical Therapeutic Chemical (ATC) system developed and maintained by WHO, and the number of DDDs can be used to express the consumption of that drug. The number of DDDs prescribed is calculated by multiplying the quantity of each dosage form prescribed by the strength of each dosage form, then dividing by the DDD value (the DDD for oral ciprofloxacin is 1 g).

### Secondary care dataset

Secondary care data were accessed via https://hospitalmedicines.genomium.org/,^22^ which in turn extracts data from the NHSBSA SCMD dataset for England.^23^ We extracted data on ciprofloxacin and levofloxacin, as well as comparator agents with similar spectrums of activity: co-amoxiclav, piperacillin/tazobactam, co-trimoxazole, meropenem, clarithromycin, amoxicillin and ceftriaxone. As this dataset reports chemical products, we defined piperacillin/tazobactam by tazobactam sodium, co-trimoxazole by sulfamethoxazole, and amoxicillin/clavulanate by potassium clavulanate. None of these are widely used outside the antibiotic combination. Data were available from April 2019 until November 2023, at a monthly level.

We estimated the number of prescriptions by assuming a 5 day course length for all infections and assuming BNF standard dosing regimens. This led to one prescription being 5 g for ciprofloxacin, 5 g for levofloxacin, 15 g for meropenem, 15 g for amoxicillin, 5 g for clarithromycin, 5 g for ceftriaxone, 8 g for co-trimoxazole (assuming 800 mg per dose of sulfamethoxazole), 3 g for co-amoxiclav (assuming 200 mg per dose of clavulanate, and IV therapy), and 7.5 g for piperacillin/tazobactam (assuming 500 mg per dose of tazobactam). We recognize these are assumptions and are simply produced to aid comparison with primary care prescribing.

### Hospital data

From December 2020 until December 2023, NHS England produced monthly hospital utilization data. These are in the format of number of occupied bed days per month and form part of the Urgent and Emergency Care Situation Report.^24^

### Analytic approach

As there have been numerous warnings from multiple drug regulators including the FDA, MHRA and EMA over this time, and as the interventions are likely not applied instantaneously, we took the approach of visualizing and quantifying change over time, rather than performing an analysis at a specific timepoint. Specific timepoint approaches have been shown to be highly sensitive to statistical methods and/or timepoints chosen.^25^ Our datasets largely overlap, although the first major MHRA warnings and restriction occurred in March 2019, prior to our secondary care dataset.

For this reason, and because of the increased potential for potentially less appropriate prescribing occurring in primary care (due to the reduced severity of illness and reduced burden of bacterial infection, and less use of fluoroquinolones in guidelines), we focus most of our analysis on the primary care data but include the secondary care data for completeness.

We performed linear regression for each drug/drug class with date as the exploratory variable, with the scale set to evaluate changes in prescriptions per year. Absolute prescription rates were calculated with respect to the total English population in the given year. To aid visualization, we scaled some analyses so prescriptions on the first study month were the reference, and report figures as a percentage change. As regional data (from ICBs) were available for primary care data, we also ran analyses at the ICB level.

To compare with other classes/antibiotics, we performed a Z-test for the beta and standard error of the linear regression for each class.^26^ For aid of visualization, we also performed linear regression of ratios (e.g. the ratio of co-amoxiclav to ciprofloxacin over calendar time).

Finally, for the time where it was available, we analysed data adjusted for inpatient population. That is, we divided the number of prescriptions by the number of inpatients for each month to generate prescriptions/month/patient.

### Software and codes

We used R 4.31 for analyses, and used the tidyverse package for data wrangling and plotting.^27^ All code is available at https://github.com/gushamilton/floroquinolone, and the analysis is completely replicable by running the same code locally. Regression was performed using the linear regression model in R, while some plots fitted splines via ggplot.

### Ethics

All data used in this analysis are publicly available. No ethics approval was therefore required.

## Results

**Figure 1.**
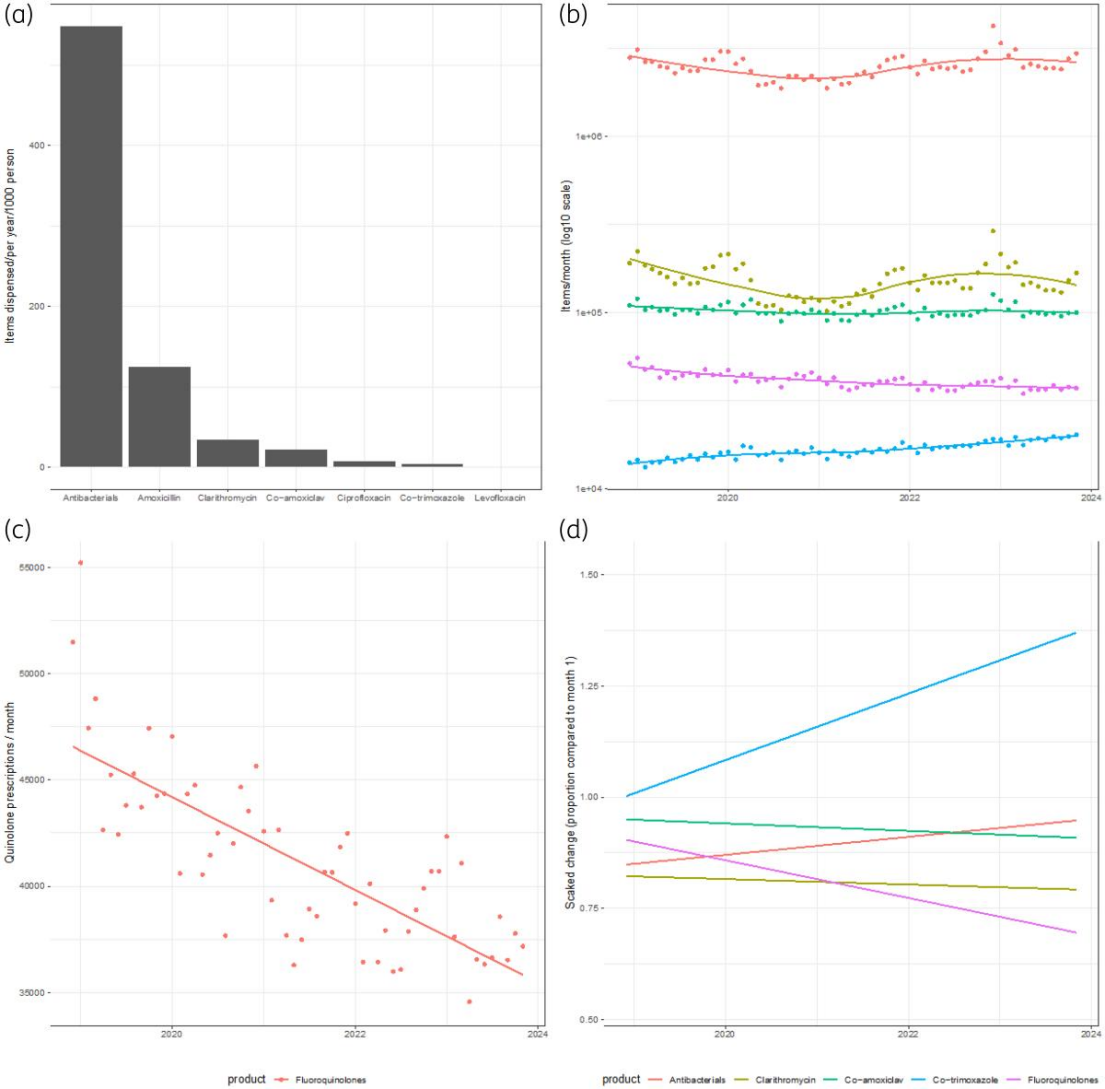
(a) Items dispensed per year per 1000 population for a variety of antimicrobials and antimicrobial classes over the whole study period. (b) Change over time in prescription of various antimicrobials: clarithromycin, co-amoxiclav, co-trimoxazole and fluoroquinolones. All antibacterial usage over this time is plotted. (c) Just fluoroquinolones shown to help visualize the change. (d) The same as (b) but rescaled so each drug class starts at 1. Linear regression lines are shown with 95% CIs.

Our primary analysis focused on primary care prescribing, where the risk–benefit ratio of fluoroquinolone prescribing is most questioned. Across the study period (∼5 years), 151 144 401 prescriptions for antibacterial agents were given across England, with an estimated population of 59.6 million (2021 census data^28^). This equates to 549 prescriptions per year/1000 people. Approximately one-fifth of this was made up of amoxicillin (125 prescriptions per year per 1000 population). Fluoroquinolones represented a small proportion of antibiotics prescribed (9 per year per 1000 population). This is visualized in Figure 1(a).

Over the study period, antibacterial prescription was static [Figure 1(b); +2% change per year, 95% CI −0.56% to 4.6%; *P* = 0.12]. However, fluoroquinolone prescription decreased substantially over time [−4.2%, 95% CI −5.2% to −3.3%; *P* = 6 × 10^−13^; Figure 1(c)]. This was driven by ciprofloxacin reduction, which was 20 times more common than levofloxacin prescription. Figure 1(d) shows these changes scaled so the first month was set to 1 for all products. This change in fluoroquinolone prescribing was offset by a large increase in co-trimoxazole prescribing (change per year +7.5%, 95% CI 6.5%–8.5%; *P* = 4.4 × 10^−22^). No other tested antimicrobial changed substantially. Table 1 shows all estimates, with Z-tests comparing change in trends versus all fluoroquinolones. This showed the trend in fluoroquinolone prescription was reduced compared with all other antimicrobials.

**Table 1.** Estimates of the change in prescribing per year of each agent across the primary care study period

Agent	Change per year, % (95% CI)	*P* value	*P* value for Z-test against trend for all fluoroquinolones^a^
Ciprofloxacin	−5.1 (−6 to −4.2)	4.0 × 10^−16^	8.9 × 10^−2^
Fluoroquinolones	−4.2 (−5.2; −3.3)	6.2 × 10^−13^	NA
Co-amoxiclav	−0.84 (−2.1; 0.37)	1.7 × 10^−1^	3.8 × 10^−6^
Clarithromycin	−0.61 (−3.9; 2.7)	7.1 × 10^−1^	1.8 × 10^−2^
Overall	2% (1.2–2.7)	3.7 × 10^−6^	2.0 × 10^−25^
Antibacterials	2 (−0.56 to 4.6)	1.2 × 10^−1^	2.2 × 10^−6^
Amoxicillin	2.5 (−2.4 to 7.4)	3.1 × 10^−1^	3.5 × 10^−3^
Penicillins	2.8 (−0.72 to 6.2)	1.2 × 10^−1^	4.9 × 10^−5^
Levofloxacin	3.8 (1.7–5.8)	5.7 × 10^−4^	7.3 × 10^−13^
Co-trimoxazole	7.5 (6.5–8.5)	4.4 × 10^−22^	9.1 × 10^−69^

^a^Z-test relative to fluoroquinolone coefficient, calculated as per Clogg *et al.*^26^ This compares whether there is a difference between these two estimates (i.e. a difference in trend between the two agents).

**Figure 2.**
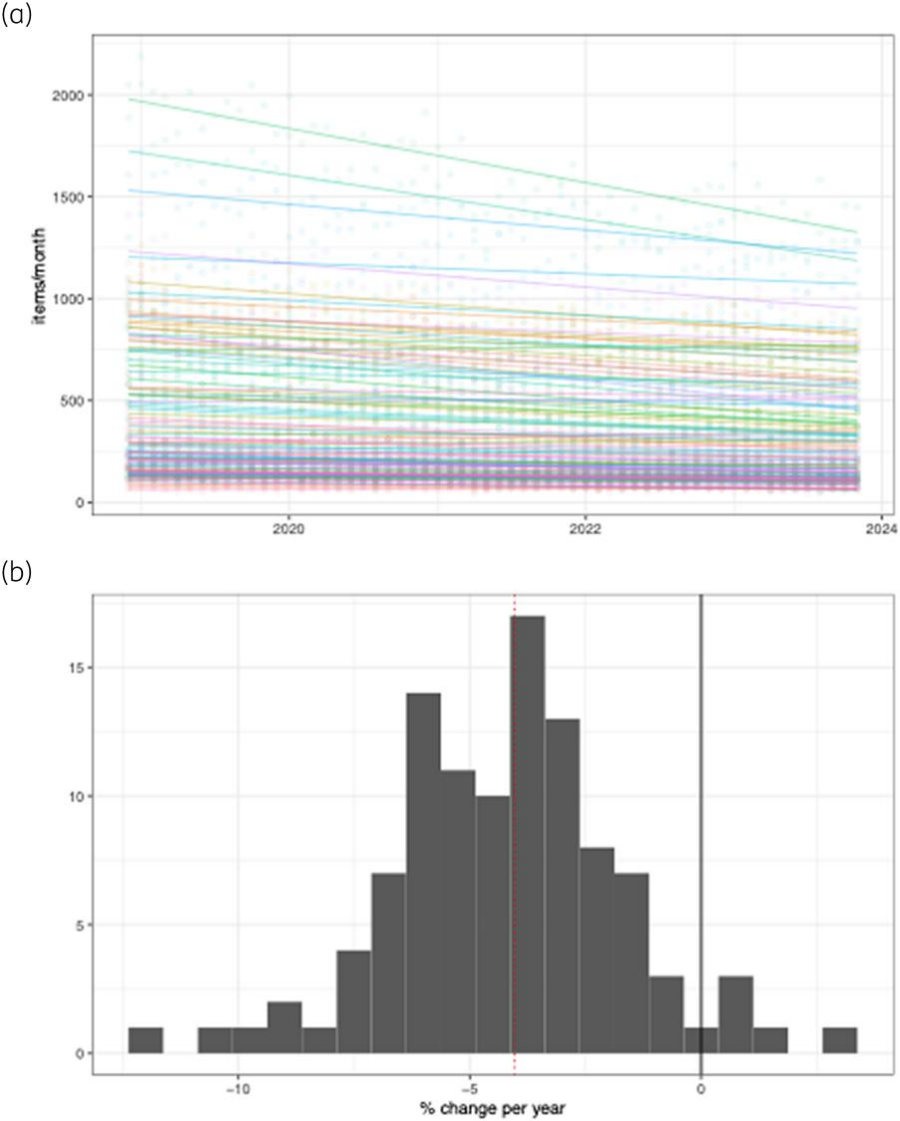
(a) Changes in prescribing of fluoroquinolones across ICBs. Multiple linear regression lines across all 101 ICBs are fitted. Each line represents an ICB, with the faded dots representing prescribing at that given month. (b) Change per year for each ICB plotted as a histogram. The black line represents no change per year, while the dotted red line represents the overall change per year in England.

There are 101 ICBs in England. Over this period, only five care boards reported an increase in fluoroquinolone usage, with only one (West Leicestershire, +3.1%, 95% CI 1.5%–4.7%; *P* = 2.8 × 10^−4^) being measured with any statistical confidence [Figure 2, Table S1 (available as Supplementary data at *JAC* Online)]. In contrast, the majority of ICBs had large reductions in fluoroquinolone usage, with 37% of ICBs reducing their prescription by 5% or more per year. The biggest reduction came from NHS Oldham ICB, with a reduction in fluoroquinolone usage by −12% per year (95% CI −65% to −47%; *P* = 1 × 10^−23^).

**Figure 3.**
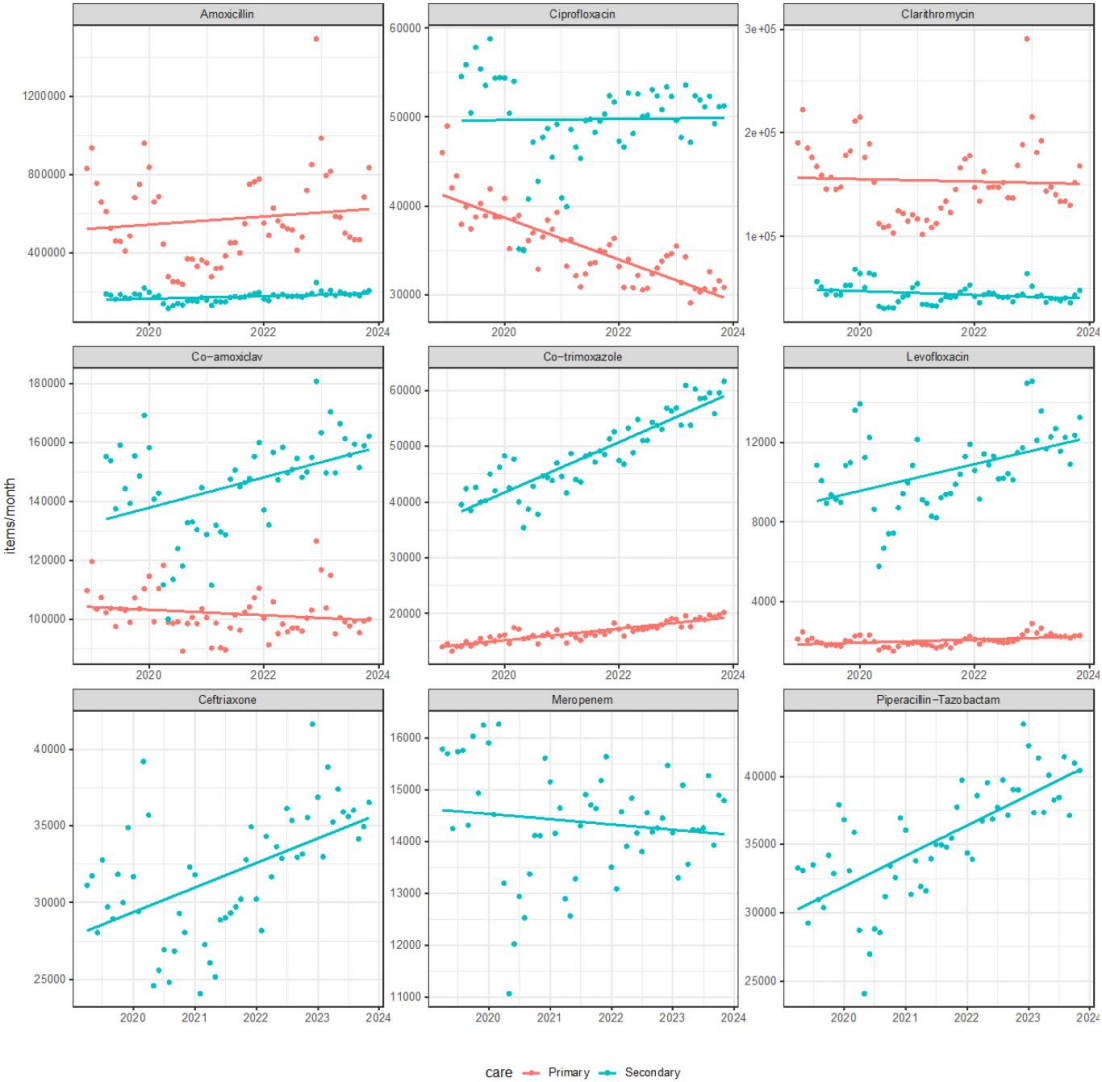
Prescribing (items/month) of various antimicrobial agents in primary and secondary care. Data taken from different sources and transformations applied, so interpret raw comparisons with caution. Linear regression with 95% CIs fitted.

We then analysed data in secondary care. This was slightly more challenging as the weight of each product in grams is provided, and then this was converted (assumptions in Methods section) to estimate and compare with the primary care data. In Figure 3, we compared estimates for multiple antimicrobial classes in primary and secondary care. Fluoroquinolone usage was generally higher in secondary care (around 50% higher total prescriptions), in contrast to amoxicillin and clarithromycin, which were much more common in primary care. Co-trimoxazole usage was much higher in secondary care. There was much more variability in secondary care prescribing, particularly in early 2020, likely related to variability in hospital occupancy rates and the cancellation of much elective surgery during the COVID-19 pandemic. Unlike in primary care, there were no clear trends identified for ciprofloxacin, clarithromycin or amoxicillin prescriptions over time in secondary care. However, there was increasing usage of co-amoxiclav, co-trimoxazole, ceftriaxone, levofloxacin, and piperacillin/tazobactam over this period (all *P* < 0.001, Table 2).

**Table 2.** Relative rates of prescribing of various antimicrobial agents in secondary care

Agent	% Change per year, % (95% CI)	*P* value	*P* value for Z-test against ciprofloxacin^a^	*P* value for Z-test against levofloxacin^a^
Co-trimoxazole	11 (9.9–13)	4.3 × 10^−21^	1.1 × 10^−22^	1.4 × 10^−3^
Tazobactam	6.7 (5–8.4)	1.3 × 10^−10^	3.3 × 10^−8^	3.9 × 10^−1^
Levofloxacin	6.2 (3–9.3)	2.3 × 10^−4^	3.6 × 10^−4^	5.0 × 10^−1^
Ceftriaxone	5.1 (2.9–7.3)	1.9 × 10^−5^	1.8 × 10^−4^	2.9 × 10^−1^
Amoxicillin	3.7 (1.4–6)	2.5 × 10^−3^	7.1 × 10^−3^	1.0 × 10^−1^
Co-amoxiclav	3.3 (1.4–5.2)	8.1 × 10^−4^	6.3 × 10^−3^	5.7 × 10^−2^
Ciprofloxacin	0.11 (−1.7 to 1.9)	9.0 × 10^−1^	5.0 × 10^−1^	3.6 × 10^−4^
Meropenem	−0.65 (−2 to 0.72)	3.5 × 10^−1^	2.5 × 10^−1^	3.2 × 10^−5^
Clarithromycin	−3.2 (−6.3 to −0.11)	4.3 × 10^−2^	3.1 × 10^−2^	9.8 × 10^−6^

The second column provides the percentage change per year, while the last two columns compare whether the trend is different to the ciprofloxacin or levofloxacin trend.

^a^Z-test relative to ciprofloxacin coefficient, calculated as per. Clogg *et al.*^26^ This compares whether there is a difference between these two estimates (i.e. a difference in trend between the two agents).

Performing a Z-test comparing the regression coefficients, we identified evidence that all these agents had increased prescribing relative to ciprofloxacin prescribing (Z-score *P* values in Table 2). These data suggest that although ciprofloxacin usage has been stable in secondary care (+0.11%, 95% CI −1.7% to 1.9%), the usage of comparable agents with similar spectrums of activity has increased relative to ciprofloxacin. Levofloxacin usage did increase over this time period (+6.2%, 95% CI 3%–9.3%; *P* = 2 × 10^−4^), but again, relatively less co-trimoxazole and piperacillin/tazobactam (both *P* < 0.001), and with a similar increase to ceftriaxone.

**Figure 4.**
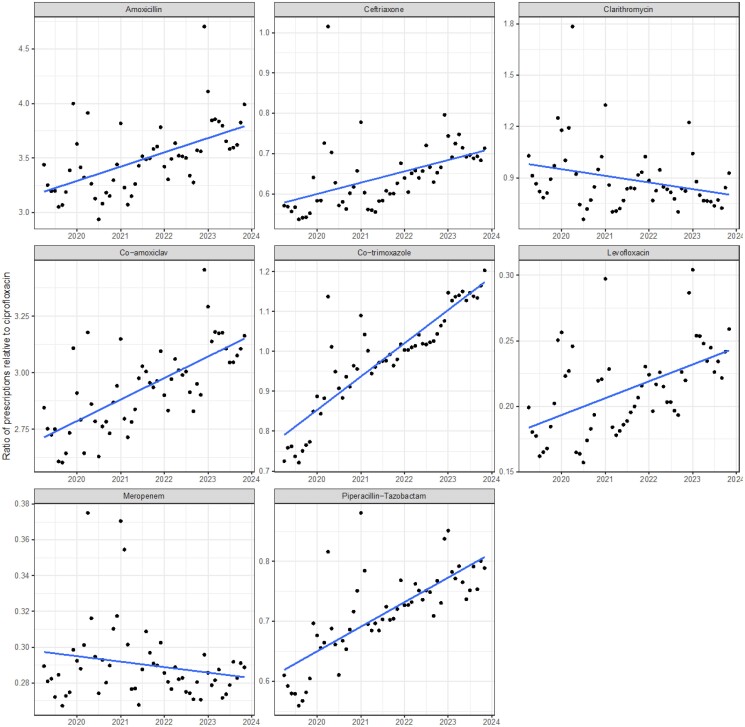
The relative ratio of prescription of each of the antibacterial agents in secondary care, as compared with ciprofloxacin, over time. Linear regression fitted with 95% CIs.

To aid comparison, a final plot showing the ratio of prescribing relative to ciprofloxacin is shown in Figure 4. This shows that over the study period, the relative prescribing of ciprofloxacin has fallen. At the start of 2020, approximately three prescriptions containing amoxicillin were given for each ciprofloxacin prescription; by late 2023, this was nearly four. This trend is shown for all tested agents except meropenem, clarithromycin and levofloxacin, which had a relative increase compared with ciprofloxacin (although small on the absolute terms) of 0.36% per year.

**Figure 5.**
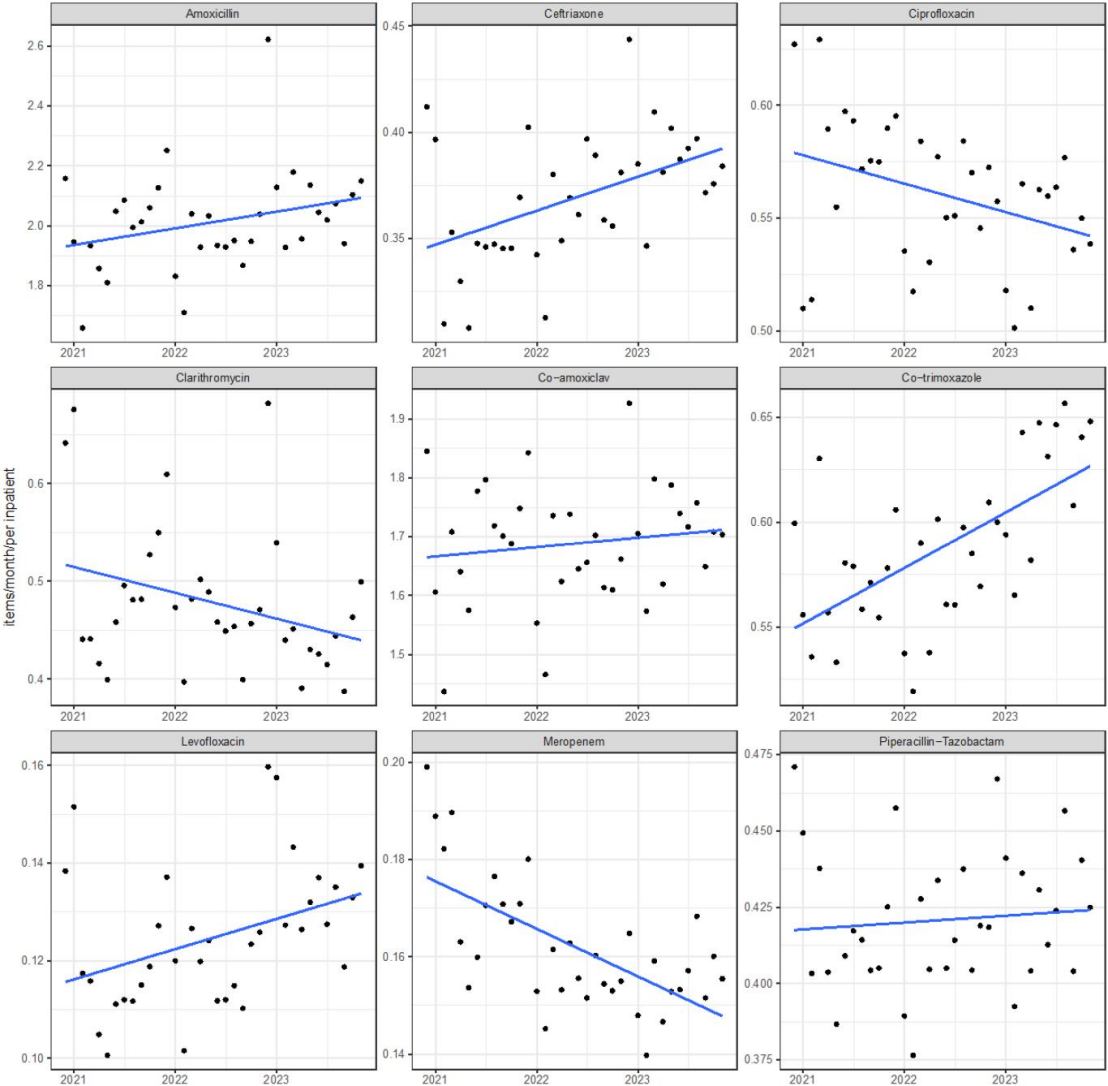
Prescriptions of each agent, adjusted for hospital utilization, so the *y*-axis is on the scale of prescriptions per inpatient per month. Linear regression (blue line) fitted with 95% CI (grey area).

Finally, from December 2020, we had access to monthly bed occupancy from NHS England and used this to adjust and generate estimated prescriptions per admitted person per month. This period starts >1 year after the MHRA and EMA warnings, and inspection of Figure 3 shows an already steep drop in ciprofloxacin prescribing between 2020 and 2021 of around 15%, which would not be captured in this analysis. Between December 2020 and December 2023, there was a large rise in occupied beds, from around 78 000 to 92 000 inpatients (∼15% increase, Figure 1). When adjusting for hospital bed utilization, we identified a reduction in ciprofloxacin (but not levofloxacin) usage, with a −2% reduction in prescribing per year (95% CI −3.9% to −0.15%; *P* = 0.04, Figure 5). Given the steep drop prior to this time period, this analysis shows that relative to hospital utilization, reductions in secondary care prescribing are similar, although slightly smaller, than in primary care for ciprofloxacin. In contrast, levofloxacin prescription did increase (+4.5%, 95% CI +0.63% to +8.3%), meaning overall fluoroquinolone prescriptions were stable (−0.8%, 95% CI −2.5% to +0.8%).

## Discussion

Our work uses both primary and secondary care data for the whole of England to explore fluoroquinolone prescriptions over the last 5 years and assess the influence of EMA/MHRA restrictions on prescribing. We identified clear reductions in primary care prescribing of ciprofloxacin, which accounted for the vast majority of all primary care fluoroquinolone prescriptions. This was in contrast to usage of comparator antibiotics that were largely static.

In secondary care, antimicrobial prescribing varied much more, likely due to the COVID-19 pandemic. Absolute ciprofloxacin prescribing was stable across the time period. However, comparator antibiotic prescriptions increased, consistent with a relative reduction in ciprofloxacin prescribing. For the time period from December 2020 onwards (by which time an ∼15% reduction in ciprofloxacin prescribing had occurred), we had hospital utilization data. When adjusting for this, relative ciprofloxacin usage reduced by around 2% per year. This is consistent with increased pressure and workload on the NHS in England, while maintaining relatively infrequent use of fluoroquinolones compared with other agents. Levofloxacin prescription did, however, increase. These data are similar to published analyses in primary care during the pandemic, where a reduction in fluoroquinolone prescription was noted but an increase in those for penicillins and other antibiotics.^29^

One major reason for the continued use of fluoroquinolones in secondary care is that there are remarkably few good oral options for the therapy of some infections. Potential agents often require IV administration, and there is also pressure to reduce the use of certain other agents (e.g. third-generation cephalosporins, β-lactam/β-lactamase inhibitors and carbapenems). Resistance rates to co-trimoxazole and co-amoxiclav in Gram-negatives are ∼30%, with ciprofloxacin at only 17% in the recent English Surveillance Programme for Antimicrobial Utilisation and Resistance (ESPAUR) report.^30^ Additionally, some randomized trial data and meta-analyses, particularly in pneumonia, suggest that fluoroquinolones are superior to other options in this condition, which is the main reason for antimicrobial prescribing in acute admissions.^31^ Other indications for fluoroquinolones may be relatively uncommon in terms of proportional numbers of patients affected, but by their nature require prolonged courses for up to 3–6 months, for example prosthetic joint or vascular implant infection.

As such, it is not surprising that there has been some resistance to the MHRA’s guidance. The Scottish Antimicrobial Prescribing Group (SAPG) response to the MHRA advice supports the ‘restrictive’ use of fluoroquinolones, but noted the potential for ‘unintended consequences’ of the MHRA advice.^32^ Specifically, the MHRA advice may ‘inadvertently promote the selection of broader-spectrum and IV’ antibiotics and ‘delay or prevent IV-to-oral switch’. Similar issues were raised in a *BMJ* news article^18^ and an opinion piece by the authors of this paper.^17^

As far as we are aware, the only other study examining post EMA/MHRA fluoroquinolone prescribing trends is by Ly *et al*.,^19^ funded by the EMA and analysing UK data from 2016 to 2020. This study used a multisegmented regression approach to identify monthly percentage changes in relation to specific communications from regulators. Although this study is reported in the MHRA Drug Safety Update as showing ‘no change’ in prescribing, it does show a reduction in fluoroquinolone prescribing, and is estimated by Ly *et al*.^19^ to be ‘at best, a reduction in prescriptions of around 25%’. As such, our results are concordant and extend the study period and show further reductions since then. The ESPAUR report for 2023 provides supportive data, with quinolone prescription dropping from 0.565 in 2018 to 0.456 in 2022 (*P* = 0.04).^30^ the United Kingdom Health Security Agency Fingertips data also provide similar results, with largely static total antibiotic prescribing but a reduction in broad-spectrum use over the last 5 years.^33^ Interestingly, a study of all antimicrobial prescribing over a longer period (2014–24), identified a reduction in overall antibiotic prescribing, whereas our data suggested largely static antimicrobial prescribing.^34^

Limitations of our study include its entirely ecological focus. We had no access to individual-level data, and so we can only focus on trends without deeper insight. Interpretation of our secondary care data was reliant on transformation of product weight into assumed product courses, although these transformations would only change absolute differences between comparator antibiotics, and not changes in trend. Our use of hospitalization data as a proxy for hospital pressure is imperfect, and only reflects total inpatients (not, for example, admissions). Given fluoroquinolones are used in both acute admissions and inpatients, it would be challenging to accurately model hospital pressure, and inpatient hospitalization is a reasonable proxy.

In summary, national prescribing data for England show an approximate 5% reduction per year in ciprofloxacin prescribing in primary care, where there is the most concern about ‘inappropriate’ prescribing of this drug class. This is clearly a large, statistically precise, and important reduction, which has continued over the 5 year period; however, the exact reasons for this reduction cannot be determined from these data.

## Supplementary Material

dkae440_Supplementary_Data

## Supplementary data

Figure S1 and Table S1 are available as Supplementary data at *JAC* Online.
