# Supplementary material for: Trends in fluoroquinolone prescribing in UK primary and secondary care between 2019 and 2023
Source: J Antimicrob Chemother. 2024 Dec 21;80(2):518–27. doi: 10.1093/jac/dkae440 (PMC11787900; doi:10.1093/jac/dkae440)
Supplement: dkae440_Supplementary_Data [file dkae440_supplementary_data.zip › Supplementary Figures.docx]

Supplementary Figures:

**Figure S1**: Bed utilisation in English NHS trusts from December 2020 until December 2023. Data from NHS England.
